# Seawater and Sunlight as Critical Ageing Factors Affecting the Mechanical Performance of Knitted Swimwear Fabrics

**DOI:** 10.3390/ma18235346

**Published:** 2025-11-27

**Authors:** Gabriela Vanja, Vesna Marija Potočić Matković, Ivana Salopek Čubrić

**Affiliations:** Department of Textile Design and Management, University of Zagreb Faculty of Textile Technology, Prilaz baruna Filipovića 28 a, 10000 Zagreb, Croatia; marija.potocic@ttf.unizg.hr (V.M.P.M.); ivana.salopek@ttf.unizg.hr (I.S.Č.)

**Keywords:** swimwear, polyamide, polyester, yarn, ageing, seawater, sun, tensile properties

## Abstract

This study investigates the effects of seawater and sunlight ageing on the structural and mechanical properties of knitted fabrics designed for swimwear. Nine fabrics with varying polyamide, polyester, and elastane ratios in yarn were subjected to 200 h seawater exposure, with and without sunlight, followed by washing and drying cycles to simulate real training and use conditions. The evaluated properties included mass per unit area, thickness, horizontal and vertical density, bursting strength, breaking force, and breaking elongation. Results showed a slight increase in mass and thickness after ageing, reflecting fabric shrinkage in the course direction. Breaking force decreased on average after ageing, with statistically significant reductions in the wale direction under combined seawater and sunlight exposure, whereas shrinkage occasionally produced apparent strengthening effects. Breaking elongation increased in the wale direction and decreased in the course direction, though without statistical significance. Correlation analysis revealed that ageing alters the dependence of mechanical properties on fabric mass per unit area and thickness, with seawater enhancing strength in the wale direction, while sunlight shifted the effects toward the strength in the course direction. These findings demonstrate that seawater and sunlight are critical ageing factors that affect swimwear performance, emphasising the need for their inclusion in durability assessments.

## 1. Introduction

Swimwear has historically served two main purposes: leisure and competition. From the heavy wool bathing dresses of the 19th century to the rubberised silk and jersey knitted fabrics of the 1920s, the goal was always to reduce resistance and improve speed. The 1930s saw the introduction of Lastex, adding much-needed elasticity, while the 1960s popularised nylon, and additionally introduced bold prints and crochet styles. Throughout the 20th century, swimwear evolved at the intersection of sport, fashion, and advances in synthetic fibres such as nylon, shaping modern designs [1,2]. With further technological progress, by the late 1990s fabrics were developed that offered lower hydrodynamic resistance. Since swimmers expend roughly 90% of their energy countering drag, manufacturers have increasingly prioritised materials and designs that minimise resistance [3].

Like other garments, swimwear are expected to maintain its performance during use; however, due to its specific purpose, it is exposed to severe external factors that accelerate ageing, whether in sport or leisure. Polymer ageing involves irreversible changes caused by light, radiation, oxygen, heat, and other factors, often acting together and amplifying the overall effect. It manifests as loss of ductility, strength, impact resistance, polymer mass, and visible changes in appearance [4]. Long-term exposure to solar radiation, temperature, humidity, ozone, and impurities leads to photochemical degradation and oxidation of macromolecules, the main drivers of deterioration [5]. Because these parameters interact, understanding their influence is crucial for evaluating performance and limiting damage.

Polyamide (PA), polyester (PES), and elastane (EL) have been widely used in swimwear, largely due to the suitability of their physical and mechanical properties [2]. PA 6 and 6.6 offer excellent wear resistance and balanced mechanical properties but absorb moisture, which can alter performance. They are sensitive to acids (hydrolysis) and UV light (chain scission) [6]. PES, produced by condensation like PA, is widely used in textiles. Compared to PA, they absorb less water and show higher dimensional stability but also undergo UV-induced chain scission and hydrolysis in the presence of water [6,7].

Extensive research has investigated the effects of UV radiation, water, temperature, humidity, and chemical exposure on polymer materials [6,8,9,10]. A late 1980s study [11] on swimwear knitted fabric (85% PA 6.6, 15% EL) dyed with acid dyes showed that exposure to chlorinated and seawater accelerated degradation more than xenon light alone. The extent of degradation varied with colour, with violet samples degrading the most and navy the least. In the work of Potočić Matković et al. [12], swimwear knitted fabric (PA/PES with EL) were exposed to chlorinated water under shade or sunlight for 200–300 h. Only slight increases in mass per unit area and thickness were observed, but fabrics aged for 300 h in chlorine and sunlight exhibited a pronounced decrease in breaking force (up to approximately 66%) and reduced moisture management, with sunlight further intensifying degradation. Another experimental study [3] examined nine sports swimsuit knitted fabrics with different PA/PES–EL ratios, aged for 100 h in seawater and sun. All samples exhibited changes, including reduced breaking force, increased elongation, and alterations in mass per unit area, thickness, water absorption, water spreading, and drying time. Fabrics with higher EL content (59% PA, 41% EL) were less affected in terms of mass per unit area and thickness. More recently, Salopek Čubrić et al. [13] developed ageing protocols for PA and PES swimwear under indoor and outdoor pool conditions. After ageing, washing, and drying, the materials were evaluated for durability and comfort through microscopy, tensile tests, and fluid transport measurements. The results revealed surface fibrillation, up to 40% reduction in breaking force, increased elongation, and more pronounced negative effects from outdoor ageing compared to indoor conditions.

PA and PET microfibers are among the most abundant microplastics in the marine environment [14,15,16]. Their degradation depends on polymer properties and environmental conditions, with UV radiation accelerating plastic breakdown, micro- and nanoplastic formation, and toxic compound leaching [17,18]. Delre et al. [19] suggested that 7–22% of all floating plastic released to the sea could already be photodegraded, indicating that photodegradation may represent a key sink for floating plastic. According to research [18], in seawater under simulated sunlight, PA fibres showed mainly surface changes without fragmentation after 56 days, whereas PET fibres exhibited both surface degradation and fragmentation. Additive chemicals leached from both polymers, some of which may persist and contribute to marine pollution. Cüreklibatır Encan [20] examined the effects of UV ageing and seawater on warp-knitted swimwear fabrics after 120 and 240 h of artificial weathering. Breaking force, elongation, elasticity, stiffness, and air permeability were significantly reduced, while SEM revealed micro-damages without chemical changes in FTIR spectra. UV radiation was identified as the main deteriorating factor, whereas seawater had minimal influence.

Although several studies have examined the degradation of polyamide and polyester swimwear under UV radiation, chlorinated water, or simulated seawater, most of them were conducted under artificial ageing conditions using xenon lamps or controlled chambers. Only limited research has explored real outdoor marine environments, where the combined influence of natural sunlight, seawater composition, and weather variability can produce different degradation pathways. This study addresses this gap by developing and applying a novel accelerated ageing protocol that exposes swimwear fabrics to natural seawater and a combination of seawater and sunlight for 200 h, followed by standardised washing and drying cycles to simulate realistic use. By testing nine commercially relevant PA/EL and PES/EL fabrics, it provides comparative data on the durability and dimensional stability of materials currently used in swimwear production. The findings of the study are expected to enhance understanding of how exposure to realistic marine ageing conditions influences fabric performance.

## 2. Materials and Methods

### 2.1. Materials

For this study, a set of nine knitted fabrics was selected, consisting of blends of PA and EL yarns in varying proportions and differing in their masses per unit area, with one exception containing PES and EL yarns. These materials, specifically intended for the manufacture of swimwear, are characterised by durability, resistance to chlorine, elasticity in both principal directions ensuring high comfort, and medium-to-strong compression to provide optimal muscle support. Three samples share the same PA/EL ratio of 80/20. All materials are marked with the letter S (abbreviation for swimming) followed by an ordinal number. The tested fabrics were commercially sourced from well-known Italian manufacturers. All fabrics were warp-knitted structures with a standard UV-protection finishing treatment applied during production. Table 1 provides an overview of the materials, their identification codes, yarn composition, and knitted fabric parameters, confirming their representativeness of materials commonly available on the market for swimwear applications.

### 2.2. Methods of Measurement

In the experimental part, the following physical and mechanical properties of the fabrics were examined: horizontal and vertical density, mass per unit area, thickness, breaking force, breaking elongation, and bursting strength.

Mass per unit area was determined according to method 5 in ISO 3801:1977 [21]. For this purpose, circular samples with a surface area of 100 cm^2^ were cut and weighed on an analytical balance Kern ALJ 220-4 (Kern & Sohn GmbH, Balingen-Frommern, Germany) with an accuracy of ±0.001 g. Fabric thickness was measured using the DM-2000 thickness tester (Wolf Messtechnik GmbH, Freiberg, Germany) in accordance with EN ISO 5084:1996 [22] under a pressure of 1 kPa. During testing, a pressure of 1 kPa was applied to the surface of the specimen to ensure standardised measurement conditions. Horizontal and vertical densities, that are used to describe and observe the structure of the fabric, were measured on 100 × 100 mm samples using a Dino-Lite PRO HR microscope (200×) (Dino-Lite Europe, Almere, The Netherlands) under controlled conditions (20 ± 2 °C, 65 ± 2% RH). Loops were counted over 10 mm in course and wale directions, with measurements repeated at 10 positions for accuracy. Tensile force at break was tested according to ISO 13934-1:2013 [23] using the Statimat M tensile tester (Textechno, Mönchengladbach, Germany). Test specimens measuring 200 × 50 mm were prepared in both the course and wale directions. During testing, a constant tensile force was applied at a speed of 100 mm/min until specimen rupture. For each fabric, five specimens were tested in both loop directions to ensure result reliability, and values of breaking force and elongation at break were recorded. Bursting strength was assessed according to ISO 13938-2:2019 [24] on circular specimens (50 mm in diameter) subjected to a steel ball, with the applied force measured until fabric rupture.

Statistical analysis was performed using TIBCO Statistica 14.0.0 (Cloud Software Group, Fort Lauderdale, FL, USA, 2020). Since the same set of nine samples (S1–S9) was analysed before and after treatment, a paired *t*-test was applied (n = 9). Given that n < 30, the data were treated as small samples. Prior to performing the *t*-test, the normality of the data distribution was verified using the Kolmogorov–Smirnov (K–S) test. The significance level was set at α = 0.05. Based on these conditions, hypotheses for the *t*-test were formulated to assess differences between paired measurements.

Correlation analysis was also performed using TIBCO Statistica 14.0.0 (Cloud Software Group, 2020). A correlation matrix was generated, and Pearson correlation coefficients were calculated to evaluate linear relationships between variables. Marked correlations were considered statistically significant at *p* < 0.05. Pearson coefficients range from –1 to +1, indicating the strength and direction of the linear relationship.

### 2.3. Ageing Method

The accelerated ageing protocol for swimwear materials was developed by the authors of this study. In this study, seawater ageing conditions were applied. The protocol consisted of immersing the samples in seawater for 200 h under sunlight, followed by 10 washing and drying cycles. All samples were immersed outdoors in seawater during summer season in Mediterranean climate zone, at 42°38′25″ N latitude and 18°06′30″ E longitude. Samples were immersed in containers filled with natural seawater collected from the nearby coast. For sunlight exposure, the containers were placed in a sunlit area of a seaside terrace, while for the non-sunlight condition, identical containers were kept under a roofed shaded area, protected from direct solar radiation. This setup allowed controlled comparison of seawater exposure with and without the influence of sunlight, under the same ambient temperature and humidity conditions. The maximum daily air temperatures during this period ranged from 32.2 to 33.5 °C, while the minimum ranged from 27.0 to 28.3 °C, with no precipitation. The average daily relative humidity was between 46% and 61%. Weather data were provided by the Croatian meteorological and hydrological service. Samples were exposed to seawater under natural sunlight for approximately 14–15 h per day, corresponding to typical summer daylight duration on the Adriatic coast, and were rinsed and air-dried overnight between exposure periods. The UV irradiance was not directly measured; however, during the summer period in the Dubrovnik region, the average UV index typically ranges between 7 and 9, with peak values around 10 at midday (data based on the Croatian Meteorological and Hydrological Service).

Seawater used for ageing was collected on the Adriatic coast near Dubrovnik. On-site physicochemical parameters were not measured at the time of sampling; therefore, representative values from national Adriatic monitoring programmes were adopted. According to these data, surface salinity in the central and southern Adriatic is typically 38–39‰, with surface pH around 8.0, and an ionic composition characteristic of open seawater (Cl^−^, Na^+^, SO_4_^2−^, Mg^2+^, Ca^2+^, K^+^).

After 200 h of seawater immersion, the samples were washed in a washing machine using a short programme (30 min at 30 °C) with ECE Formulation Non-Phosphate Reference Detergent (without optical brighteners, James Heal, Halifax, UK), applied at a dosage of 5 g per 1 kg of textile. Following each wash, the samples were air-dried in the shade. The washing procedure was repeated 10 times. Ten machine washing cycles were selected to simulate the effects of occasional machine washing that swimwear fabrics typically undergo during regular summer use.

## 3. Results and Discussion

The results of the study are presented as the results of a range of physical–mechanical properties of knitted fabrics as follows:

- Mass per unit area;
- Thickness;
- Horizontal and vertical density;
- Breaking force in the direction of wales and courses;
- Breaking elongation in the direction of wales and courses.

### 3.1. Fracture Morphology

The representative microscopic images presented in Table 2, captured at 200× magnification, show the knitted swimsuit materials in their non-aged state and after 200 h of immersion in seawater under shaded conditions and direct sunlight, followed by ten washing and drying cycles. In the non-aged state (sample S8 shown as representative), the loop structure is uniform, compact, and vertically aligned, with densely packed filaments and no visible signs of deterioration.

After 200 h of ageing in shade and seawater, the first signs of surface modification become evident. Localised fibrillation appears across the yarn surface, reflected in short protruding fibres, while the knitted loops remain largely intact. These changes indicate the onset of structural weakening despite the absence of UV radiation.

More pronounced degradation is observed after exposure to direct sunlight. Fibrillation becomes widespread, with longer and more numerous protruding filaments, partially fractured fibres, and a less clearly defined loop geometry. The reduction in the inner loop space further indicates fabric shrinkage. Such behaviour is consistent with the synergistic effects of UV radiation, seawater, and mechanical stresses on PA materials.

Overall, both ageing conditions induce changes in surface morphology; however, deterioration is significantly more severe after sunlight exposure. The increased fibrillation and structural disruption are expected to adversely affect the breaking elongation and breaking force of the fabrics, although no catastrophic failure of the loop structure was observed.

### 3.2. Changes in Mass per Unit Area After Ageing

**Figure 1:**
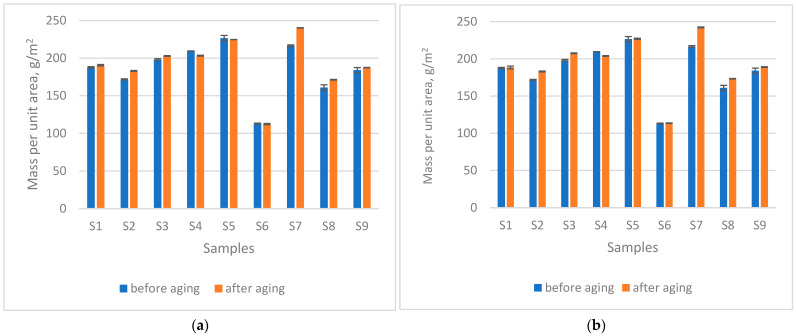
Mass per unit area after (**a**) 200 h seawater exposure, (**b**) 200 h seawater + sun exposure.

Changes in mass per unit area after ageing (Figure 1) show a slight tendency toward an increase, from an average of 2.8% (after 200 h seawater exposure) to 3.6% (after 200 h seawater + sun exposure). This effect can be attributed to the minor shrinkage of the material under different ageing conditions. A *t*-test comparison of the samples before and after ageing without sun exposure, at a significance level of α = 0.05 (*p* = 0.1136), indicates that soaking in seawater for 200 h did not cause statistically significant changes. Similarly, no statistically significant difference was observed after 200 h of combined exposure to sun and seawater (*p* = 0.0646). Under both performed types of the exposure fabrics to outdoor natural weathering, the increase in mass per unit area was observed for the materials S1–S3 and S7–S9. Such behaviour is in line with the studies reported in [3,12,25]. A slight increase in mass per unit area that was observed may indicate swelling or surface roughening of fibres due to water absorption and photochemical changes. This could in turn influence fibre integrity and potentially affect fibre shedding or microplastic release during subsequent washing or wear. Further research using appropriate analytical approaches is needed to confirm this assumption, making the assessment of microplastic release an important topic for future studies.

### 3.3. Changes in Thickness After Ageing

**Figure 2:**
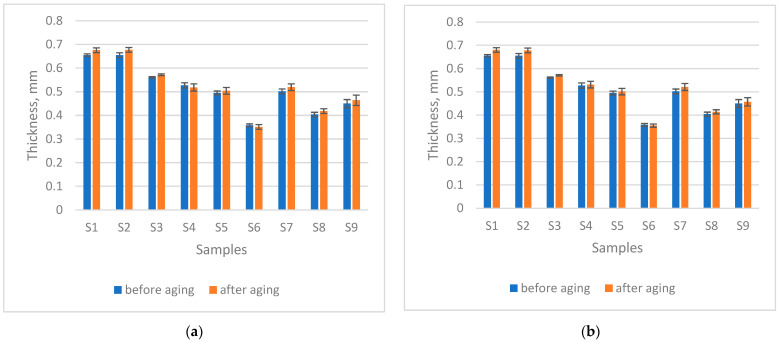
Thickness after (**a**) 200 h seawater exposure, (**b**) 200 h seawater + sun exposure.

Changes in thickness after ageing (Figure 2) indicate a slight increase, from an average of 1.85% (after 200 h seawater exposure) to 2.0% (after 200 h seawater + sun exposure), suggesting an alteration in the knitted loop structure under different ageing conditions. A *t*-test comparison of the samples before and after ageing without sun exposure, at a significance level of α = 0.05 (*p* = 0.0252), confirmed that immersion in seawater for 200 h caused a statistically significant increase in material thickness. Moreover, a statistically significant difference was also observed after 200 h of combined exposure to sun and seawater (*p* = 0.0068). Previous studies [26,27,28] have shown that the local thickening of PES fibres during ageing is linked to annealing, during which polymer chains undergo rearrangement, resulting in structural changes. This molecular relaxation and redistribution lead to an increase in fibre thickness and diameter. The results of this study showed a slight decrease in thickness for the fabric made of PES fibres (sample S4) after 200 h of seawater exposure. However, after 200 h of combined seawater and sunlight exposure, the thickness of this material increased, which is consistent with previous research and highlights the stronger influence of sunlight on fibre thickening compared to seawater exposure alone.

### 3.4. Changes in the Coefficient of Loop Density After Ageing

To better interpret the results for material thickness and mass per unit area, additional measurements of fabric structural properties were conducted by evaluating the horizontal (Dh) and vertical (Dv) densities. These parameters also serve as indicators of knitted fabric shrinkage in the wale and course directions after ageing. The values of horizontal and vertical densities before and after ageing are presented in Table 3. Samples S1–S4 and S5–S9 display comparable vertical and horizontal densities, yet differ, as previously noted, in mass per unit area and thickness. The results further reveal that vertical densities vary across the tested materials, generally showing a decrease in the number of loops after the ageing process. Conversely, most materials exhibit an overall increase in horizontal density, confirming shrinkage in the course direction. A *t*-test comparison of the samples before and after ageing without sun exposure, at a significance level of α = 0.05 (*p* = 0.2374 for vertical density and *p* = 0.1926 for horizontal density), indicates that 200 h of seawater immersion did not cause statistically significant changes. Similarly, no significant differences were observed after 200 h of combined sun and seawater exposure (*p* = 0.1378 for vertical density and *p* = 0.1739 for horizontal density).

The coefficient of loop density (C) was determined for the knitted fabric both before and after ageing and represents the overall loop density of the knitted fabric [29]. It was calculated using Equation (1):

(1) C=Dh/Dv

where

Dh—horizontal knitting density (loops/10 mm),

Dv—vertical knitting density (loops/10 mm).

Changes in the coefficient of loop density indicate a shift in the balance between horizontal and vertical densities, reflecting greater shrinkage in one direction than the other. The largest changes were observed after 200 h of seawater ageing in samples S9 (30.00%) and S5 (21.88%), leading to dimensional instability and, ultimately, alterations in swimsuit shape. The increase in loop density in these samples, which initially contained the highest EL content, suggests damage to the EL fibres.

### 3.5. Changes in Breaking Force After Ageing

**Figure 3:**
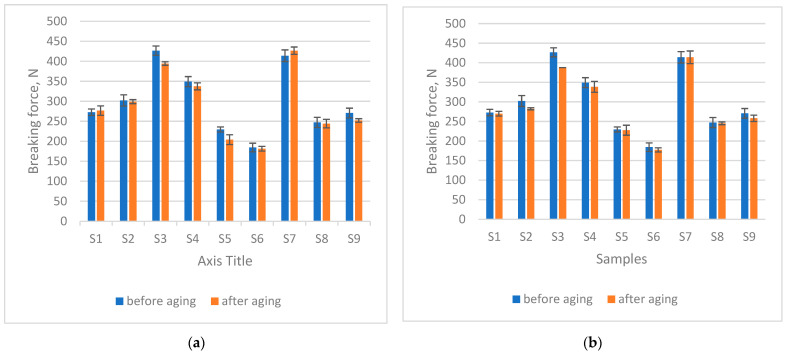
Breaking force (measured using the bursting test) after (**a**) 200 h seawater exposure, (**b**) 200 h seawater + sun exposure.

The detrimental influence of UV radiation and seawater on the breaking force of knitted fabrics is clearly demonstrated in Figure 3. After 200 h of ageing, swimwear materials exhibited a reduction in breaking force (on average, −3.13% for 200 h seawater and −3.32% for 200 h seawater + sun). Seven out of nine samples showed a decrease, whereas sample S1 displayed a slight increase of 1.43%. Sample S7 showed the most pronounced increase (+12.76%), which corresponded to one of the highest rises in mass per unit area and thickness, attributed to shrinkage. This shrinkage likely contributed to the apparent strengthening effect, although the material itself was presumably also degraded by UV radiation and seawater. In both ageing conditions, sample S3 proved to be the most sensitive, exhibiting a decrease in breaking force of −39.24%. A *t*-test comparison of the samples before and after ageing without sun exposure, at a significance level of α = 0.05 (*p* = 0.1006), confirmed that 200 h of immersion in seawater did not cause statistically significant changes. In contrast, a statistically significant difference was observed after 200 h of combined exposure to sun and seawater (*p* = 0.0330). It should be noted that the study [28] investigated polymer bottles, which differ from textile fibres in morphology, processing methods, and molecular characteristics. PET bottles typically exhibit higher intrinsic viscosity and molecular weight than PET fibres [30]. Nevertheless, the decrease observed under combined exposure in the present study corresponds to previous findings [28], which reported a reduction in the tensile strength of polymers after prolonged sunlight exposure, even when the overall shape of the materials remained unchanged. Together, these observations confirm that UV exposure—rather than water alone—is a critical factor driving the weakening of polymer-based materials, underscoring the importance of incorporating sunlight into realistic ageing protocols for swimwear fabrics.

**Figure 4:**
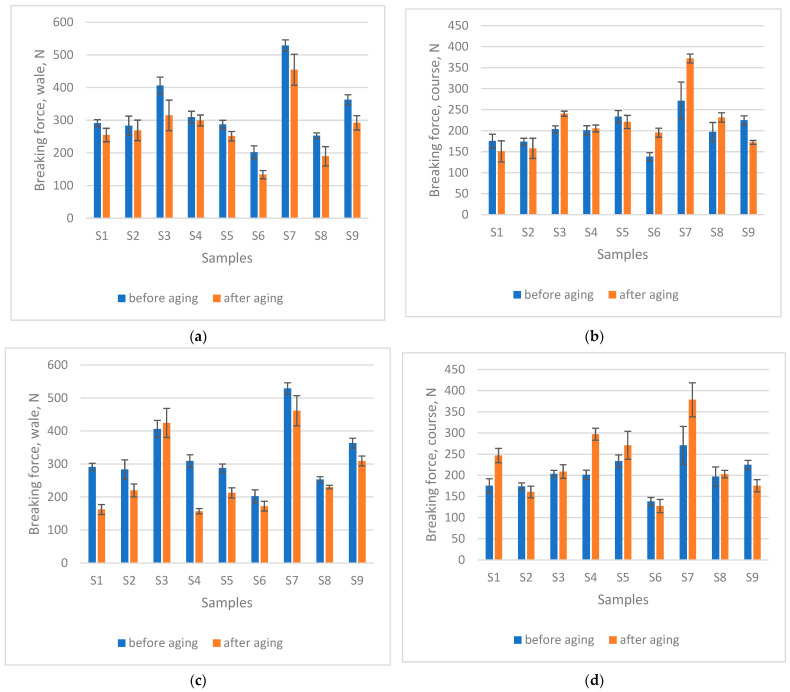
Breaking force (measured using the strip test) after 200 h seawater exposure (**a**) in the direction of wale, (**b**) in the direction of course; and after 200 h seawater + sun exposure (**c**) in the direction of wale, (**d**) in the direction of course.

The breaking force results of the knitted fabrics are presented in Figure 4a,c (wale direction) and Figure 4b,d (course direction). After 200 h of ageing, all swimwear materials exhibited a decrease in breaking force in the wale direction (average decrease of 16.48% for 200 h seawater and 21.0% for 200 h seawater + sun). Exposure to seawater caused a statistically significant reduction in breaking force in the wale direction (*p* = 0.0006), whereas no statistically significant change was observed in the course direction (*p* = 0.3938). Similarly, after 200 h of combined exposure to sun and seawater, a statistically significant decrease occurred in the wale direction (*p* = 0.0063), while the course direction again showed no significant difference (*p* = 0.1598). In some materials, however, breaking force increased, most likely as a result of shrinkage—particularly in the course direction—which contributed to an apparent strengthening effect after ageing. These findings of a significant reduction in breaking force are consistent with previous studies on the effects of seawater on PA [31,32,33] and PES [31]. In particular, artificial weathering simulating morning dew followed by sunlight gradually reduces tensile strength in PET fabrics, as chain scission produces shorter chains that can no longer form crystalline segments, leading to a decrease in crystallinity [33].

Analysis of the correlation matrix (Table 4) revealed distinct shifts in the relationship between mass per unit area and thickness of the fabric and mechanical properties under different ageing conditions. Before ageing, breaking force in the wale direction correlated moderately with mass per unit area, while thickness showed only a minor effect. In contrast, course direction strength was strongly related to mass per unit area, indicating that course strength increased with fabric mass. After 200 h of seawater exposure, the influence of mass per unit area and thickness on wale strength became more pronounced. Course strength, however, lost its earlier association with mass per unit area. These results suggest that seawater ageing enhances the mass dependence of wale strength, while course strength becomes less influenced by fabric mass per unit area. When fabrics were exposed to sunlight in addition to seawater, the dependence of breaking force in the wale direction with mass per unit area and thickness weakened considerably. Interestingly, sunlight exposure reinforced the relationship between course direction strength and mass per unit area, as well as other structural factors such as thickness.

In conclusion, the study demonstrates that ageing conditions significantly alter the relationships between fabric mass and thickness and mechanical properties. Initially, breaking forces in the wale and course directions show distinct correlations with fabric mass per unit area. Seawater ageing enhances the dependence of wale strength on fabric mass per unit area, while diminishing the influence of it on course strength. However, additional sunlight exposure modifies these trends, weakening the wale direction’s reliance on mass per unit area but strengthening the role of fabric mass per unit area and thickness in determining course strength. This evidence highlights how different environmental ageing processes distinctly impact fabric performance by altering key mechanical–structural correlations.

### 3.6. Changes in Breaking Elongation After Ageing

**Figure 5:**
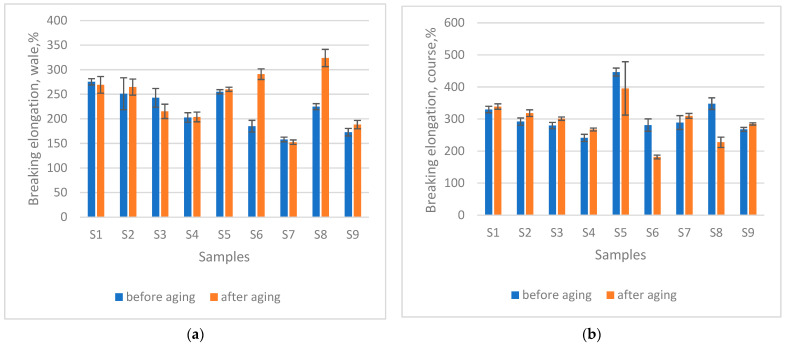

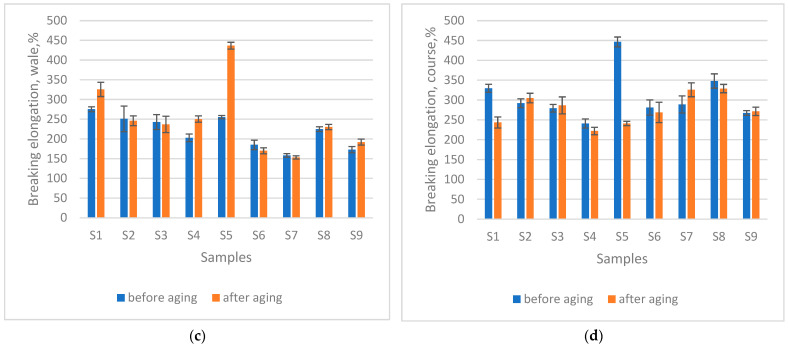
Breaking elongation after 200 h seawater exposure (**a**) in the direction of wale, (**b**) in the direction of course; and after 200 h seawater + sun exposure (**c**) in the direction of wale, (**d**) in the direction of course.

In the wale direction, ageing was associated with an increase in breaking elongation, averaging 11.22% after 200 h in seawater and 12.27% after combined seawater and sun exposure (Figure 5). These results indicate a tendency toward lower strength and material damage under ageing conditions. Nevertheless, the *t*-test indicated that the observed changes were not statistically significant, either for seawater ageing (*p* = 0.1944) or sun exposure (*p* = 0.1766).

Conversely, the course direction showed a slight reduction in breaking elongation, with average decreases of 4.17% after 200 h in seawater and 7.61% after combined seawater and sun ageing (Figure 5). Although these findings point to a minor loss of extensibility, the *t*-test again revealed no statistically significant influence of ageing in seawater (*p* = 0.4166) or sun exposure (*p* = 0.2396).

The correlation matrix between breaking elongation and material properties before and after ageing is presented in Table 5 in order to observe the influence of fabric mass per unit area and thickness on the changes in breaking elongation over the time. The analysis of the correlation matrix of breaking elongation in the wale and course directions with material properties before ageing showed only a weak dependence on mass per unit area and breaking force, while in the wale direction a moderate positive correlation with thickness was observed. After 200 h of exposure to seawater, elongation in the wale direction exhibited moderately negative correlations with mass per unit area. In contrast, elongation in the course direction showed strong positive correlations with mass per unit area and moderately with thickness. Following 200 h of combined seawater and sun exposure, elongation in both the wale and course directions displayed much weaker correlations with all material properties compared with seawater exposure alone, as seen in Table 5.

These findings indicate that ageing significantly modifies the relationships between breaking elongation and mass per unit area and thickness. Specifically, seawater ageing intensified these associations, resulting in strong positive correlations in the course direction and moderately negative correlations in the wale direction, whereas the combined effect of seawater and sun exposure diminished the overall strength of correlations, yielding weaker and less consistent relationships.

## 4. Conclusions

This study addressed the research gap in the field by developing and applying a new and more realistic accelerated ageing protocol that exposes swimwear fabrics to natural seawater and sunlight, followed by standardised washing and drying cycles to simulate realistic use. In contrast to previous studies that mainly relied on artificial laboratory ageing or isolated stress factors, this approach replicated real environmental conditions, rep-resenting a novel contribution to the field. By testing commercially relevant fabrics, it provided comparative data on the durability and dimensional stability of materials currently used in swimwear production. The study’s results indicate that exposure to seawater and sunlight leads to a slight but significant increase in mass per unit area and thickness, indicating shrinkage of the fabric. Additionally, a clear decrease in breaking force was observed under the combined influence of seawater and sunlight, suggesting structural weakening. These changes imply that swimwear fabrics may degrade faster during use, potentially shortening product lifespan, increasing the frequency of replacement, and negatively impacting sustainability by generating more textile waste and increasing the risk of fibre shedding and microplastic release. Correlation analysis showed that ageing modifies how mechanical properties depend on fabric mass and thickness, with seawater enhancing wale strength and sunlight shifting the effect toward course strength. Overall, the results of the study highlight that both seawater and sunlight are critical environmental factors that contribute to the degradation of swimwear fabrics.

## Figures and Tables

**Table 1:** Overview of swimwear materials and main fabric parameters.

Fabric ID	Yarn Parameters				Knitted Fabric Parameters	
	Type of Polymer	Yarn Ratio in a Fabric, %		Polymer Fineness, dtex	Mass per Unit Area g/m^2^	Thickness, mm
		Basic Polymer	EL			
S1	PA	80	20	110	187.84	0.655
S2	PA	80	20	62	171.75	0.655
S3	PA	78	22	150	198.13	0.561
S4	PES	78	22	150	209.59	0.527
S5	PA	59	41	44	226.46	0.495
S6	PA	73	27	33	113.11	0.358
S7	PA	80	20	88	216.52	0.502
S8	PA	80	20	88	216.52	0.502
S9	PA	72	28	44	160.97	0.404

**Table 2:** Microscopic images of the materials (a) before ageing, after (b) 200 h seawater exposure, (c) 200 h seawater + sun exposure.

Fabric ID	Before Ageing	After Ageing (200 h Seawater Exposure)	After Ageing (200 h Seawater + Sun Exposure)
S8	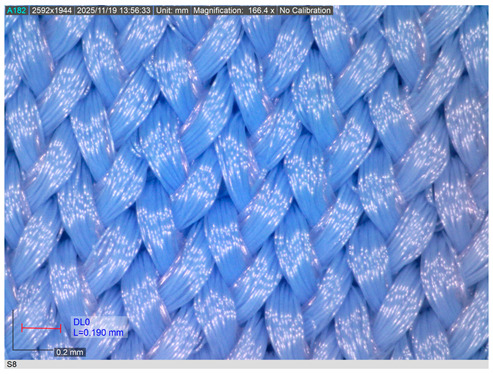	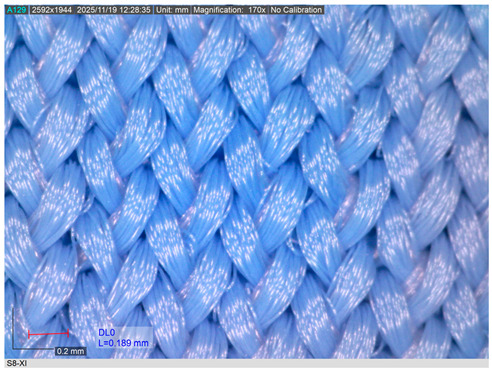	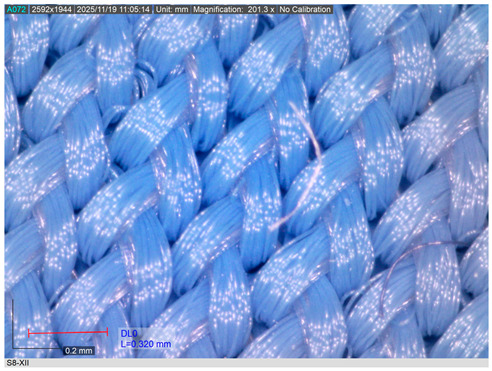
	(**a**)	(**b**)	(**c**)

**Table 3:** Results of the measured horizontal and vertical loop densities, as well as the calculated values of the loop density coefficient, obtained before and after ageing.

Fabric ID	Before Ageing		After Ageing (200 h Seawater Exposure)		After Ageing (200 h Seawater + Sun Exposure)	
	Horizontal/Vertical Loop Density (Loops/10 mm)	Coefficient of Loop Density	Horizontal/Vertical Loop Density (Loops/10 mm)	Coefficient of Loop Density	Horizontal/Vertical Loop Density (Loops/10 mm)	Coefficient of Loop Density
S1	26/46	0.57	26/44	0.59	27/42	0.64
S2	26/42	0.62	26/44	0.62	25/44	0.57
S3	26/44	0.59	27/46	0.59	27/43	0.63
S4	25/42	0.60	27/44	0.61	26/44	0.59
S5	24/26	0.92	27/24	1.13	26/24	1.08
S6	26/28	0.93	24/24	1.00	25/24	1.04
S7	24/20	1.20	24/20	1.20	24/20	1.20
S8	26/28	0.93	26/27	0.96	26/26	1.00
S9	20/26	0.77	23/23	1.00	24/24	1.00

**Table 4:** Correlation matrix between breaking force and material properties before and after ageing.

Before Ageing	Mass per Unit Area	Thickness
Breaking force wale	0.624356	0.202509
Breaking force, course	0.799983	−0.001445
**After 200 h Seawater Exposure**		
Breaking force, wale	0.840819	0.388155
Breaking force, course	0.518455	−0.241238
**After 200 h Seawater + Sun Exposure**		
Breaking force, wale	0.536134	0.009142
Breaking force, course	0.821512	0.206545

**Table 5:** Correlation matrix between breaking elongation and material properties before and after ageing.

Before Ageing	Mass per Unit Area	Thickness
Breaking elongation, wale	0.140391	0.630667
Breaking elongation, course	0.263975	−0.028645
**After 200 h Seawater Exposure**		
Breaking elongation, wale	−0.636582	−0.154641
Breaking elongation, course	0.770763	0.665606
**After 200 h Seawater + Sun Exposure**		
Breaking elongation, wale	0.315845	0.344177
Breaking elongation, course	0.021943	−0.124835

## Data Availability

The original contributions presented in this study are included in the article. Further inquiries can be directed to the corresponding author.

## References

[1] Foster L., James D., Haake S. (2012). Influence of full body swimsuits on competitive performance. Procedia Eng..

[2] Lloyd K. (2015). An Investigation into the Potential for Thermochromic Colorant Application in Women’s Swimwear. Ph.D. Thesis.

[3] Krstović K., Matković V.M.P., Čubrić I.S., Čubrić G. (2022). Physical-mechanical properties of aged knitted fabric for swimsuits. Tekstilec.

[4] Markovičová L., Zatkalíková V. (2019). The effect of UV ageing on structural polymers. IOP Conf. Ser. Mater. Sci. Eng..

[5] Mallick P.K. (2007). Fiber-Reinforced Composites: Materials, Manufacturing, and Design.

[6] McKeen L.W. (2019). The Effect of UV Light and Weather on Plastics and Elastomers.

[7] Shin M., Kim H., Kim S., Kim H.J., Oh D.X., Park J. (2024). Biodegradation behavior of polyesters with various internal chemical structures and external environmental factors in real seawater. Polym. Test..

[8] Kashi S., De Souza M., Al-Assafi S., Varley R. (2019). Understanding the effects of in-service temperature and functional fluid on the ageing of silicone rubber. Polymers.

[9] Długa A., Bajer D., Kaczmarek H. (2022). Photochemical and thermal stability of bionanocellulose/poly(vinyl alcohol) blends. Polymers.

[10] Kiersnowska A., Fabianowski W., Koda E. (2020). The influence of the accelerated ageing conditions on the properties of polyolefin geogrids used for landfill slope reinforcement. Polymers.

[11] Epps H.H. (1987). Degradation of swimwear fabrics: Effects of light, sea water and chlorine. Cloth. Text. Res. J..

[12] Potočić Matković V.M., Salopek Čubrić I., Krstović K. (2024). The impact of chlorinated water and sun exposure on the durability and performance of swimwear materials. Polymers.

[13] Salopek Čubrić I., Čubrić G., Potočić Matković V.M. (2021). Behavior of polymer materials exposed to ageing in the swimming pool: Focus on properties that assure comfort and durability. Polymers.

[14] Liu J., Liu Q., An L., Wang M., Yang Q., Zhu B., Ding J., Ye C., Xu Y. (2022). Microfiber pollution in the Earth system. Rev. Environ. Contam. Toxicol..

[15] Yang H., Chen G., Wang J. (2021). Microplastics in the marine environment: Sources, fates, impacts and microbial degradation. Toxics.

[16] Lei X., Cheng H., Luo Y., Zhang Y., Jiang L., Sun Y., Zhou G., Huang H. (2021). Abundance and characteristics of microplastics in seawater and corals from reef region of Sanya Bay, China. Front. Mar. Sci..

[17] Jansen M.A.K., Andrady A.L., Bornman J.F., Aucamp P.J., Bais A.F., Banaszak A.T., Barnes P.W., Bernhard G.H., Bruckman L.S., Busquets R. (2024). Plastics in the environment in the context of UV radiation, climate change and the Montreal Protocol: UNEP Environmental Effects Assessment Panel, Update 2023. Photochem. Photobiol. Sci..

[18] Sørensen L., Groven A.S., Hovsbakken I.A., Del Puerto O., Krause D.F., Sarno A., Booth A.M. (2021). UV degradation of natural and synthetic microfibers causes fragmentation and release of polymer degradation products and chemical additives. Sci. Total Environ..

[19] Delre A., Goudriaan M., Morales V.H., Vaksmaa A., Ndhlovu R.T., Baas M., Keijzer E., de Groot T., Zeghal E., Egger M. (2023). Plastic photodegradation under simulated marine conditions. Mar. Pollut. Bull..

[20] Cüreklibatır Encan B. (2024). Impact of artificial weathering on swimwear fabric. Fibers Polym..

[21] (1977). Textiles—Woven Fabrics—Determination of Mass per Unit Length and Mass per Unit Area.

[22] (1996). Textiles—Determination of Thickness of Textiles and Textile Products.

[23] (2013). Textiles—Tensile Properties of Fabrics—Part 1: Determination of Maximum Force and Elongation at Maximum Force Using the Strip Method.

[24] (2019). Textiles—Bursting Properties of Fabrics—Part 2: Pneumatic Method for Determination of Bursting Strength and Bursting Distension.

[25] Salopek Čubrić I., Potočić Matković V.M., Skenderi Z. (2014). Changes of the knitted fabric properties due to exposure to outdoor natural weathering. J. Eng. Fiber Fabr..

[26] Čubrić I.S., Čubrić G., Katić Križmančić I., Kovačević M. (2022). Evaluation of changes in polymer material properties due to ageing in different environments. Polymers.

[27] Šaravanja A., Dekanić T., Pušić T., Volmajer Valh J. (2023). The effect of accelerated ageing simulation on the properties of polyester fabrics. Tekstil.

[28] Chaisupakitsin M., Chairat-Utai P., Jarusiripot C. (2019). Degradation of polyethylene terephthalate bottles after long sunlight exposure. Songklanakarin J. Sci. Technol..

[29] Sait S.T., Sørensen L., Kubowicz S., Vike-Jonas K., Gonzalez S.V., Asimakopoulos A.G., Booth A.M. (2021). Microplastic fibers from synthetic textiles: Environmental degradation and additive chemical content. Environ. Pollut..

[30] Farah S., Kunduru K.R., Basu A., Domb A.J., Visakh P.M., Liang M. (2015). Molecular Weight Determination of Polyethylene Terephthalate. Poly (Ethylene Terephthalate) Based Blends, Composites and Nanocomposites.

[31] Yamano N., Kawasaki N., Ida S., Nakayama A. (2019). Biodegradation of polyamide 4 in seawater. Polym. Degrad. Stab..

[32] Le Gac P.Y., Arhant M., Le Gall M., Davies P. (2017). Yield stress changes induced by water in polyamide 6: Characterization and modeling. Polym. Degrad. Stab..

[33] Asadi H., Uhlemann J., Stranghoener N., Ulbricht M. (2021). Artificial weathering mechanisms of uncoated structural polyethylene terephthalate fabrics with focus on tensile strength degradation. Materials.

